# Alcohol consumption patterns among men who have sex with men in major cities of Myanmar: A cross-sectional study

**DOI:** 10.12688/f1000research.25707.2

**Published:** 2021-01-05

**Authors:** Kyaw-Min Htut, Chitlada Areesantichai, Myo-Myo Mon

**Affiliations:** 1 Department of Medical Research, Ministry of Health and Sports, Yangon, Myanmar; 2 College of Public Health Sciences, Chulalongkorn University, Bangkok, Thailand; 3 Drug Dependence Research Centre, WHO Collaborating Centre for Research and Training in Drug Dependence, Health and Social Science and Addiction Research Unit, College of Public Health Sciences, Chulalongkorn University, Bangkok, Thailand

**Keywords:** Men who have sex with men, alcohol, binge drinking, Myanmar

## Abstract

**Background: **Alcohol consumption patterns vary widely across the regions of the world. Although previous studies have focused on the sexual risk behaviours among men who have sex with men (MSM), studies regarding binge alcohol drinking among MSM in Myanmar are scarce.

**Methods: **A cross-sectional study was conducted to identify the alcohol consumption patterns among MSM aged over 18 years in two major cities of Myanmar where the MSM population is higher than other regions. Purposive sampling was applied and sampling was made through Myanmar MSM network. Face-to-face interviews were conducted using a structured questionnaire. Patterns of alcohol consumption were described as frequency/percentage and mean/median as appropriate. Bivariate analysis was also done to find out the association between types of MSM and binge drinking.

**Results: **A total of 256 MSM included in the study (mean age, 27.33±7.7 years). Of 256 participants, 225 MSM had the experience of alcohol consumption in their lifetime (225/256, 87.9%). Among ever drinkers, 152 MSM consumed alcohol within three months (152/225, 67.6%). Regarding beer consumption, the highest proportions of MSM from both groups (42.8%, 36.8%) consumed 1-3 times per week. Overall, 57.2% of young MSM and 41.2% of adult MSM consumed beer together with their friends. Nearly 34% of young MSM and nearly 38% of adult MMS consumed beer at gatherings of friends. At different time periods, higher proportions of Thange (partners of MSM) had experienced of binge drinking than apwint (open) and apone (hidden) (p<0.05).

**Conclusions: **The current study identified the alcohol consumption patterns in terms of type, amount, frequency at different time periods among MSM in major cities of Myanmar. It is suggested to develop and implement alcohol control policy for MSM since the proportion of current drinkers as well as binge drinking higher among these groups.

## Introduction

Globally, alcohol is widely consumed as a beverage and for recreation and socialization. Alcohol consumption patterns vary widely across the regions, ranging from daily heavy drinking to occasional hazardous drinking ( Ennett *et al*., 2016; World_Health_Organization, 2018). Excessive alcohol use and chronic alcohol binging are associated with high morbidity and mortality ( Stockings *et al*., 2016). About 40% of global population aged over 15 years had consumed alcohol while 2.3 billion of them are current drinkers. Worldwide, 44.8% of total recorded alcohol is consumed in the form of spirits. The second most consumed type of beverage is beer (34.3%) followed by wine (11.7%) ( World_Health_Organization, 2018).

Types of alcoholic beverage varied among countries in South-East Asia region. Spirit was most common in Democratic People’s Republic of Korea (97%), India (92%), Sri Lanka (85%), Philippines (72%), Thailand (69%) and Myanmar (68%). Similarly, wine was the commonest type in Indonesia (76%), Nepal (49%), and Maldives (37%) while beer was common in Brunei Darussalam (100%), Vietnam (91%), Cambodia (88%), Singapore (70%), Timor-Leste (68%), and Malaysia (61%) ( World_Health_Organization, 2018). The most common type of alcoholic beverage in Myanmar was spirits, which was consumed by 68% of drinkers ( World_Health_Organization, 2018).

Previous studies have highlighted that hazardous alcohol drinking was common among men who have sex with men (MSM), ranging from incidences of 14% to 52%, and it was also associated with unsafe sexual behaviours ( Davis *et al.*, 2016; Herrera *et al*., 2016; Liu *et al*., 2016; Santos *et al*., 2018). Furthermore, a significant association was noted between hazardous alcohol drinking and sexually transmitted infections, including HIV. According to a study conducted in China, 14.4% of MSM reported hazardous drinking and 16.8% reported binge drinking. Hazardous or binge drinkers were associated with various risky sexual behaviours such as have multiple partnerships, pay for sex, and have condomless insertive anal intercourse ( Liu *et al*., 2016). In a study in Peru, 45% of MSM and transgender women had an alcohol use disorder. Higher incidence of condomless anal intercourse was seen among participants with alcohol use disorder (AUD). However, AUD positivity was not associated with either condomless anal intercourse or recent STI/HIV infection ( Herrera *et al*., 2016). Another study in US has documented the heavy alcohol use patterns and correlates in a diverse sample of MSM using respondent-driven sampling. It was found that prevalence of RDS adjusted weekly drinking was 24.9% and weekly binge drinking was 19.3%. Independent correlates of hazardous alcohol consumption were identified as being moderately or extremely interested in reducing alcohol use; ever receiving alcohol treatment; using ecstasy; reporting syphilis diagnosis; and having more than five male partners ( Santos *et al*., 2018).

In Myanmar, HIV is concentrated among key affected populations like MSM, whereas HIV prevalence was over 10% among them ( UNAIDS, 2015). Alcohol drinking was common among MSM, as it was with other men. Although previous studies have focused on risky sexual behaviours among MSM, few studies assessed hazardous alcohol drinking among MSM. According to a previous study in Myanmar, there was 50% of lifetime drinkers and 20% of current drinker among general men ( Oo *et al.*, 2015). Similarly, in Myanmar, we know of no studies to have reported on alcohol consumption among MSM. Therefore, current study was conducted to identify the alcohol consumption patterns and binge drinking at different time periods among men who have sex with men in Myanmar.

## Methods

### Study design, population and area

A cross-sectional study was conducted among MSM those aged more than 18 years in Yangon and Mandalay, major cities of Myanmar where MSM population is higher than other regions during June and July 2020.

***Inclusion criteria***

- Self-identifying MSM;
- MSM who had engaged in insertive or receptive anal sex or both;
- MSM who have at least six months stay in Yangon and Mandalay

***Exclusion criteria***

- MSM who are not mentally sound according to the records from the centre
- MSM who do not understand and communicate in the Myanmar language

### Sampling and sample size

Purposive sampling was applied and sampling of the MSMs was made through the Myanmar MSM network. Identification of the places for recruitment of the possible participants was made after discussion with the focal persons from the networks of MSM. There might be bias resulting from applying purposive sampling; however, attempts to reduce the bias were made by providing thorough explanations to the focal person to recruit different type of MSM from different sources, such as drop-in-centre and beauty parlours. 

By considering the estimated proportion of MSM who are current drinkers as 20% ( Oo *et al.*, 2015), to achieve a 95% confidence level and an error of 5%, the minimum required sample size becomes 246 ( Wayne, 1995).

### Data collection

Firstly, a structured questionnaire was developed in English by reviewing the literature (see *Extended data*; Htut *et al*., 2020b). Then, translation was done into Myanmar language and back translation was carried out into English by a translator who was expert in both languages and had experience of translation regarding questionnaire used in MSM related research. Training of the interviewers was done at Department of Medical Research and pre-test was done at a non-study township in Yangon Region. After receiving ethical approval, at the venues where MSMs usually gather like drop-in-centers of International Non-governmental Organizations, beauty parlors, famous beautician, moat and office of community based organization eligible participants were contacted and invited to participate in the study. After getting the informed consent, data collection was done by face-to-face interview. Strict adherence to ethical principles were ensured throughout the data collection period in order to maintain the confidentiality of the information of the study participants.

### Operational definitions

According to the local terminology in Myanmar, three groups of MSM were included in the study: Apwint or open type, Apone or hidden type and Thange ( National_AIDS_Program, 2019). Apwint or open MSM are defined as individuals born biological male but who openly express themselves femininely by dress and/or social interactions. Apone or hidden MSM are defined as individuals born biological males who may also want to express themselves femininely but may not disclose this behaviour to all segments of their social networks. Tha Nge are defined as having a masculine outward appearance but have sex with men”.

Types of alcohol beverages consumed by MSM included Beer, Wine, Whisky and Rum.

### Data management and analysis

Data entry was carried out with EpiData version 3.1 and data analysis was done with SPSS version 21. Exploratory data analysis was done to check the errors, consistencies and missing values. The number of standard drinks was calculated by volume of container in liters multiplied by the percentage of alcohol volume multiplied by 0.789 (the specific gravity volume of ethyl alcohol). Binge drinking was defined as five or more standard drinks for men in a sitting or within two hours. Descriptive statistics were shown according to the data obtained from the assessment. Patterns of alcohol consumption were described as frequency/percentage and mean/median as appropriate. Bivariate analysis using chi-squared was also done to find out the association between types of MSM and binge drinking. Level of statistical significance was set as p value of <0.05.

### Ethical considerations

The proposal was submitted to Institutional Review Board, Department of Medical Research, Myanmar (Ethics/DMR/2020/036). Written informed consent was taken from the participants after thorough explanation about the objectives of the study. Confidentiality and anonymity of the information were strictly ensured. All answer sheets and data reports were kept in locked cabinet.

## Results

A total of 256 MSM were included in the study, of whom 151 participants were residents of Yangon and 105 participants were residents of Mandalay. Mean age of MSM was 27.33±7.7 years and ranged from 18 to 57 years. Tables were presented according to the age group that was categorized as 18–24 years as young MSM and ≥25 years as adult (older) MSM. As shown in Table 1, 39.1% of 18–24 years age group and 58.9% of ≥25 years age group were “apwint” (open). Regarding their education status, 52.7% of the younger age group and 39% of the older age group had attended education up to high school level. Over 46% of young MSM and over 66% of adult MSM have regular income earning job. Median monthly income was 200,000 MMK in both groups. Over 33% of young MSM were private/government staff while over 35% of adult MSM were running their own business. Individual-level responses from each participant are available as Underlying data ( Htut *et al*., 2020a)

**Table 1.** Socio-demographic characteristics of MSM from Yangon and Mandalay (n=256).

Characteristics	Age group (n=256)	
	18–24 years n (%)	≥25 years n (%)
Type of MSM Apwint (open) Apone (hidden) Thange (partner of apwint/apone)	43 (39.1) 27 (24.5) 40 (36.4)	86 (58.9) 41 (28.1) 19 (13.0)
Education Read & write Primary school Middle school High school Graduate/University	3 (2.7) 14 (12.7) 28 (25.5) 58 (52.7) 7 (6.4)	4 (2.7) 14 (9.6) 39 (26.7) 57 (39.0) 32 (31.9)
Have income earning job No/not regular Yes, always	59 (53.6) 51 (46.4)	6 (4.1) 97 (66.4)
Monthly income (Kyats) Median (IQR) Range	200,000 (166,000) 15,000 – 900,000	200,000 (187,500) 15,000 – 2,000,000
Occupation Manual labour Private/government staff NGO/INGO staff Own business Sex work Others	31 (32.3) 32 (33.3) 8 (8.3) 16 (16.7) 6 (6.3) 3 (3.1)	18 (12.9) 27 (19.3) 31 (22.1) 50 (35.7) 10 (7.1) 4 (2.9)

Of the 256 participants, 225 had experience of alcohol consumption in their lifetime (225/256, 87.9%). Among ever drinkers, 152 had consumed alcohol within the past three months (152/225, 67.6%). The amount and frequency of different types of alcohol consumption within three months in terms of amount, frequency, with whom they drink together, time of consumption and reasons are shown in Table 2. Regarding beer consumption, the mean amount consumed was 4.1±2.5 standard drinks by young MSM and 4.5 ± 3.0 standard drinks by adult MSM (ethanol concentration of 41 and 45 grams, respectively), highest proportions of MSM from both groups (42.8%, 36.8%) consumed 1–3 times per week.

**Table 2.** Amount and frequency of different types of alcohol consumption by age group of MSM within three months.

	Age group	
	18–24 years	≥25 years
Amount of alcohol use		
Beer Mean ± SD Min – Max	(n=49) 4.1 ± 2.5 1.3 – 13.0	(n=68) 4.5 ± 3.0 0.5 – 15.6
Wine Mean ± SD Min – Max	(n=6) 6.6 ± 5.2 1.3 – 15.4	(n=5) 4.1 ± 3.4 0.5 – 7.7
Whisky Mean ± SD Min – Max	(n=14) 6.5 ± 2.9 2.5 – 10.0	(n=19) 6.8 ± 4.1 1.9 – 15.6
Frequency of alcohol use, n (%)		
Beer 1/month 2–4/month 1–3/week >3/week	(n=49) 14 (28.6) 12 (24.5) 21 (42.8) 2 (4.1)	(n=68) 24 (35.3) 19 (27.9) 25 (36.8) 0 (0.0)
Wine 1/month 2–4/month 1–3/week	(n=6) 2 (33.4) 3 (50.0) 1 (16.7)	(n=5) 2 (40.0) 0 (0.0) 3 (60.0)
Whisky 1/month 2–4/month 1–3/week >3/week	(n=14) 1 (7.1) 2 (14.3) 9 (64.3) 2 (14.3)	(n=19) 4 (21.1) 6 (31.6) 9 (47.3) 0 (0.0)

Table 3 shows the types of person that MSM drink with, the reasons and timings of different types of alcohol consumption by age group of MSM within three months. Over 57.2% of young MSM and 41.2% of adult MSM consumed beer together with their friends. A majority of younger and older MSM consumed beer in the evening or at night (96% and 94.2%, respectively). Nearly 34% of young MSM and nearly 38% of adult MMS consumed beer for the reason of a friends’ gathering.

**Table 3.** People who drink together, reason and timing of different types of alcohol consumption by age group of MSM within three months.

	Age group	
	18–24 years n (%)	≥25 years n (%)
With whom consume alcohol together		
Beer Friends MSM friends Sexual partner Alone	(n=49) 28 (57.2) 17 (34.7) 1 (2.0) 3 (6.1)	(n=68) 28 (41.2) 27 (39.7) 7 (10.3) 6 (8.8)
Wine Friends MSM friends Alone	(n=6) 2 (33.4) 4 (66.7) 0 (0.0)	(n=5) 2 (40.0) 2 (40.0) 1 (20.0)
Whisky Friends MSM friends Sexual partners Alone	(n=14) 7 (50) 6 (42.9) 0 (0.0) 1 (7.1)	(n=19) 7 (36.9) 8 (42.1) 3 (15.8) 1 (5.3)
Timing of alcohol consumption		
Beer Morning/afternoon Evening/night Whole day	(n=49) 1 (2.0) 47 (96.0) 1 (2.0)	(n=68) 3 (4.4) 64 (94.2) 1 (1.4)
Wine Evening/night	(n=6) 6 (100.0)	(n=5) 5 (100.0)
Whisky Morning/afternoon Evening/night Whole day	(n=14) 1 (7.1) 13 (92.9) 0 (0.0)	(n=19) 0 (0.0) 17 (89.5) 2 (10.5)
Rum Evening/night	(n=6) 6 (100.0)	(n=3) 3 (100.0)
Reason of alcohol consumption		
Beer Gathering Special occasion Feel unhappy Feel happy Want to use Attract sexual partner	(n=49) 17 (34.1) 1 (2.0) 7 (14.3) 9 (18.4) 12 (24.4) 3 (6.1)	(n=68) 26 (38.2) 1 (1.5) 6 (8.8) 15 (22.1) 17 (25.0) 3 (4.4)
Wine Gathering Social occasion Feel unhappy Feel happy Want to use Attract sexual partner	(n=6) 1 (16.7) 0 (0.0) 1 (16.7) 3 (50.0) 1 (16.7) 0 (0.0)	(n=5) 1 (20.0) 1 (20.0) 0 (0.0) 1 (20.0) 1 (20.0) 1 (20.0)
Whisky Gathering Special occasion Feel unhappy Feel happy Want to use Attract sexual partner	(n=14) 6 (42.9) 0 (0.0) 1 (7.1) 1 (7.1) 4 (28.5) 2 (14.3)	(n=19) 8 (42.1) 1 (5.3) 0 (0.0) 1 (5.3) 9 (47.4) 0 (0.0)

Table 4 shows that binge drinking was associated with type of MSM. At different time periods, higher proportions of Thange (partner of MSM) had experienced of binge drinking than apwint (open) and apone (hidden), and the association was statistically significant (p<0.05).

**Table 4.** Binge drinking according to the category of MSM at different time periods among MSM from Yangon and Mandalay.

	Type of MSM			p-value
	Apwint (Open) n (%)	Apone (Hidden) n (%)	Thange (Partner) n (%)	
Within one month Non-binge drinking Binge drinking	(n=53) 35 (66.0) 18 (34.0)	(n=46) 27 (58.7) 19 (41.3)	(n=41) 10 (24.4) 31 (75.6)	0.0001
Within three months Non-binge drinking Binge drinking	(n=58) 37 (63.8) 21 (36.2)	(n=51) 33 (64.7) 18 (35.3)	(n=43) 16 (37.2) 27 (62.8)	0.01
Within six months Non-binge drinking Binge drinking	(n=70) 45 (64.3) 25 (35.7)	(n=50) 33 (66.0) 17 (34.0)	(n=44) 19 (43.2) 25 (56.8)	0.04
Within one year Non-binge drinking Binge drinking	(n=82) 55 (67.1) 27 (32.9)	(n=53) 33 (62.3) 20 (37.7)	(n=48) 20 (41.7) 28 (58.3)	0.01
Lifetime Non-binge drinking Binge drinking	(n=107) 77 (72.0) 30 (28.0)	(n=63) 42 (66.7) 21 (33.3)	(n=55) 25 (45.5) 30 (54.5)	0.003

## Discussion and recommendation

Worldwide, the prevalence of alcohol consumption was 43% in general population while about half has never consumed alcohol. Of all participants in present study, nearly 90% have ever consumed alcohol in their life time and over 50% were current drinkers within one month. Previous studies have documented the alcohol consumption among general population in Myanmar. They reported the prevalence of 50% lifetime drinkers and 20% current drinkers. The proportion of ever drinkers in current study was much higher than those from previous studies done in two different townships in Myanmar ( Oo *et al*., 2015; Win & Areesantichai, 2014). These studies focused on the general adult population, which might differ from the MSM population. These differences in study population and time periods might contribute to the discrepancy. Regarding the types of alcoholic beverages, as reported in the report of World Health Organization, common types of alcoholic beverages consumed in Myanmar included spirits, beer and wine. Likewise, beer, whisky and wine were the common alcoholic beverages stated by MSM in current study.

Studies in China and other countries also highlighted alcohol consumption among MSM and general population but using different screening tools. Additionally, previous studies have documented the prevalence of alcohol consumption at different time periods ( Liu *et al*., 2016; Lu *et al*., 2019). In present study, nearly 70% of MSM had consumed alcohol within the past 3 months and 36.2% of Apwint (open type), 35.3% of Apone (hidden type) and 62.8% of Thange (sexual partners of MSM) had experience of binge drinking. In a large-scale study in China, over 56% of 3,588 MSM had consumed alcohol in the past 3 months and 17% were binge drinkers ( Liu *et al*., 2016). Though recent alcohol consumption within 3 months among MSM was similar between the studies, the proportion of binge drinkers was higher in present study.

In Peru, 45% of MSM had an alcohol use disorder and over 90% were hazardous drinkers ( Herrera *et al*., 2016). Another study in China among a general adult population aged 18 to 34 years also identified the prevalence of alcohol consumption as about 45% ( Lu *et al*., 2019). Similarly, another study in US among MSM documented that nearly 14% and 25% were monthly and weekly binge drinkers ( Santos *et al*., 2018). Unlike other studies in China, Fan *et al*. (2016) documented that 23% of MSM had consumed a drink containing alcohol in the previous year while 7% were heavy alcohol drinkers.

The current study also identified the proportion of drinkers and binge drinkers in different time periods (one month, three months, six months, one year and lifetime). and over one third to nearly two third among different types of MSM reported binge drinking within three months. A higher proportion of participants drinking alcohol was noted in current study than in previous studies among MSM population. Differences in background sociocultural conditions may contribute to this discrepancy. ( Liu *et al*., 2016; Lu *et al*., 2019). It was detected that binge drinking was more common among Thange than other two types of MSM at different time periods showing higher risk among that particular group of MSM.

There was a limitation in the present study that should be acknowledged. Alcohol consumption patterns were self-reported and their behaviours could not be validated with other methods, such as observation. However, we tried to overcome this limitation by carefully explaining the objectives of the study to allow participants to answer with accurate responses.

The development and implementation of an alcohol control policy for MSM should be considered, since over half of them were current drinkers (within one month). Between one-third and three-quarters of them had binge drinking at different time periods, which could lead to adverse health and social consequences. Emphasis should be done more on the partners of MSM as higher proportion of them had practice of binge drinking.

## Data availability

### Underlying data

Figshare: msm_data_alcohol.sav. https://doi.org/10.6084/m9.figshare.12911510.v1 ( Htut *et al*., 2020a).

This project contains the de-identified underlying data from each participant for the present study.

### Extended data

Figshare: Questionnaire_Alcohol consumption patterns among men who have sex with men in major cities of Myanmar: A cross-sectional study. https://doi.org/10.6084/m9.figshare.12926636.v1 ( Htut *et al*., 2020b).

This project contains the questionnaire used in the present study.

Data are available under the terms of the Creative Commons Attribution 4.0 International license (CC-BY 4.0).

## References

[ref-1] Davis A Kaighobadi F Stephenson R: Associations Between Alcohol Use and Intimate Partner Violence Among Men Who Have Sex with Men. * LGBT Health.* 2016;3(6):400–406. 10.1089/lgbt.2016.0057 27906642 PMC5165665

[ref-2] Ennett ST Jackson C Cole VT: A multidimensional model of mothers' perceptions of parent alcohol socialization and adolescent alcohol misuse. *Psychol Addict Behav.* 2016;30(1):18–28. 10.1037/adb0000119 26415053 PMC4752377

[ref-3] Fan W Lu R Wu G: Alcohol drinking and HIV-related risk among men who have sex with men in Chongqing, China. *Alcohol.* 2016;50:1–7. 10.1016/j.alcohol.2015.09.004 26632032

[ref-4] Herrera M Konda K Leon S: Impact of alcohol use on sexual behavior among men who have sex with men and transgender women in Lima, Peru. *Drug Alcohol Depend.* 2016;161:147–154. 10.1016/j.drugalcdep.2016.01.030 26896169 PMC4807690

[ref-5] Htut KM Areesantichai C Mon MM: msm_data_alcohol.sav. *figshare.*Dataset.2020a 10.6084/m9.figshare.12911510.v1

[ref-6] Htut KM Areesantichai C Mon MM: Questionnaire_Alcohol consumption patterns among men who have sex with men in major cities of Myanmar: A cross-sectional study. *figshare.*Journal contribution.2020b 10.6084/m9.figshare.12926636.v1 PMC7814280 33500776

[ref-7] Liu Y Ruan Y Strauss SM: Alcohol misuse, risky sexual behaviors, and HIV or syphilis infections among Chinese men who have sex with men. *Drug Alcohol Depend.* 2016;168:239–246. 10.1016/j.drugalcdep.2016.09.020 27723554 PMC5523945

[ref-8] Lu W Xu J Taylor AW: Analysis of the alcohol drinking behavior and influencing factors among emerging adults and young adults: a cross-sectional study in Wuhan, China. *BMC Public Health.* 2019;19(1):458. 10.1186/s12889-019-6831-0 31039783 PMC6492408

[ref-9] National_AIDS_Program: Myanmar Integrated Biological and Behavioral Surveillance Survey & Population Size Estimates among MSM 2015.2019 Reference Source

[ref-10] Oo WM Aung MS Soe PP: Alcohol consumption among adult males in urban area of Thanlyin Township, Yangon Region, Myanmar. *Int J Res Med Sci.* 2015;3(11):3192–3196. 10.18203/2320-6012.ijrms20151161

[ref-11] Santos GM Rowe C Hern J: Prevalence and correlates of hazardous alcohol consumption and binge drinking among men who have sex with men (MSM) in San Francisco. *PLoS One.* 2018;13(8):e0202170. 10.1371/journal.pone.0202170 30118495 PMC6097698

[ref-12] Stockings E Hall WD Lynskey M: Prevention, early intervention, harm reduction, and treatment of substance use in young people. *Lancet Psychiatry.* 2016;3(3):280–296. 10.1016/S2215-0366(16)00002-X 26905481

[ref-13] UNAIDS: Situation Analysis of the HIV Response among Men who have Sex with Men and Transgender Persons in Myanmar.2015 Reference Source

[ref-14] Wayne W: Biostatistics: A Foundation of Analysis in the Health Sciences. John Wiley & Sons. In: Inc.1995 Reference Source

[ref-15] Win SMS Areesantichai C: Pattern of Alcohol Drinking among Adults in Pha-An Township, Myanmar. *J Health Res.* 2014;28(Suppl):S41–S45. Reference Source

[ref-16] World_Health_Organization: Global status report on alcohol and health 2018: World Health Organization.2018 Reference Source

