# Supplementary material for: Alcohol consumption patterns among men who have sex with men in major cities of Myanmar: A cross-sectional study
Source: F1000Res. 2021 Jan 5;9:1149. Originally published 2020 Sep 17. [Version 2] doi: 10.12688/f1000research.25707.2 (PMC7814280; doi:10.12688/f1000research.25707.2)
Supplement: Peer review history

# Peer review: Reviewer response for version 2

**DOI:** 10.5256/f1000research.31469.r76605

**Published:** 2021-01-18

**Authors:** Myat Thuthu Win, Inn Kynn Khaing

**Affiliations:** 1 AIMST University, Bedong, Malaysia; 2 Unit of Pharmacology, Faculty of Medicine, AIMST International University, Bedong, Malaysia

1. The spelling of "Tha Nge" to "Thange" to be consistent. Still some place not updated. (Operational definitions).
2. "15-24 yrs" has been updated to "18-24 yrs".
3. Bivariate analysis using chi-squared & p- value were added.
4. Slight modification in discussion.

Is the work clearly and accurately presented and does it cite the current literature?

Partly

If applicable, is the statistical analysis and its interpretation appropriate?

Partly

Are all the source data underlying the results available to ensure full reproducibility?

Partly

Is the study design appropriate and is the work technically sound?

Yes

Are the conclusions drawn adequately supported by the results?

Partly

Are sufficient details of methods and analysis provided to allow replication by others?

Partly

Reviewer Expertise:

Diabetes, Aging.

We confirm that we have read this submission and believe that we have an appropriate level of expertise to confirm that it is of an acceptable scientific standard.

# Peer review: Reviewer response for version 1

**DOI:** 10.5256/f1000research.28371.r75393

**Published:** 2020-12-11

**Authors:** Myat Thuthu Win, Inn Kynn Khaing

**Affiliations:** 1 AIMST University, Bedong, Malaysia; 2 Unit of Pharmacology, Faculty of Medicine, AIMST International University, Bedong, Malaysia

1. It is good idea to study alcohol consumption which is very popular among men who have sex with men (MSM) but it is not clearly described why the authors want to choose target population of this group as well as aim of this study. We suggest that the prevalence of alcohol drinking among men in Myanmar should be stated in general so the prevalence in this group will be higher or not can be determined.
2. The study design is suitable and sample size is adequate. We recommend that age range should be included in abstract. (18-57 yrs) We aware that the authors used 15-24 yrs in (Table -1 ) but young MSM should be 18-24yrs (Table -1) because the study was done over 18 yrs of age in this study.
3. The authors tried to explore the data as much as possible but it is difficult to get exact data of alcohol consumption among MSM. Out of 256 participants, 225 participants consumed alcohol in their lifetime, 89.7%, but the authors did not specify about others who were more than 18 yrs of age or not. (Never drinking alcohol in their lifetime?) These data only indicates alcohol drinking but no impact. We recommend that if the authors can correlate the data with some points like the prevalence of sexually transmitted disease among them, it will be more scientific and reproducible. We also doubtful about the lowest salary among MSM is only 1,5000 MMK per month. If the salary is too low, it will affect the alcohol consumption.
4. Justify for using bivariate analysis of Binge drinking according to the category of MSM at different time periods.
  It is not relevant with Table -4 results in discussion: “over one third to nearly two third among different types of MSM reported binge drinking within three months”.
5. As we mentioned above, it is difficult to guarantee for full reproducibility. It will need to be more scientific study and outcome for reliable data. But, we understood that there would be some constraints because of COVID-19 pandemic.
6. We suggest that there should be some outcome for conclusion for alcohol consumption among MSM. After describing the results, the authors could not discuss properly about binge drinking among different category of MSM in different time periods and it does not highlight how different time periods of binge drinking have an influence on different category of MSM. So it was difficult to get conclusion from the results. It is not a good conclusion.

Is the work clearly and accurately presented and does it cite the current literature?

Partly

If applicable, is the statistical analysis and its interpretation appropriate?

Partly

Are all the source data underlying the results available to ensure full reproducibility?

Partly

Is the study design appropriate and is the work technically sound?

Yes

Are the conclusions drawn adequately supported by the results?

Partly

Are sufficient details of methods and analysis provided to allow replication by others?

Partly

Reviewer Expertise:

Diabetes, Aging.

We confirm that we have read this submission and believe that we have an appropriate level of expertise to confirm that it is of an acceptable scientific standard, however we have significant reservations, as outlined above.

# Response

**Published:** 2021-01-09

**Authors:** Kyaw-Min Htut

**Affiliations:** Department of Medical Research, Myanmar

**According to comment 1**, the prevalence of alcohol drinking among men in Myanmar had described as "According to a previous study in Myanmar, there was 50% of lifetime drinkers and 20% of current drinker among general men."

**According to the comment 2**, I have edited 18-24 yrs instead of 15-24 yrs in every sentence and tables.

**Comment number 3**: “These data only indicates alcohol drinking but no impact”

**Response: **It does not include any impact of alcohol consumption as the study does not focus and include in our study objectives.

 C **omment number 3**: “We recommend that if the authors can correlate the data with some points like the prevalence of sexually transmitted disease among them, it will be more scientific and reproducible.”

**Response: **Thanks so much for your suggestion and we agree that these points are very important to discuss. But we don’t have such kind of data because it is out of our study scope.

 C **omment number 3 of reviewer 2**: “We also doubtful about the lowest salary among MSM is only 1,5000 MMK per month. If the salary is too low, it will affect the alcohol consumption.”

**Response: **It is not the salary. We just ask about their income in terms of money they received in anyway because some of them did not have regular job.

**According to comment 4,** we did bivariate analysis between binge drinking and type of MSM since there would be difference in behaviors according to the types of MSM

 "It was detected that binge drinking was more common among Thange than other two types of MSM at different time periods showing higher risk among that particular group of MSM" and "Emphasis should be done more on the partners of MSM as higher proportion of them had practice of binge drinking" were added according to your 5th and 6th comments.

# Peer review: Reviewer response for version 1

**DOI:** 10.5256/f1000research.28371.r71570

**Published:** 2020-09-23

**Authors:** May Soe Aung

**Affiliations:** 1 Department of Preventive and Social Medicine, University of Medicine 1, Yangon, Myanmar

It is an interesting article which highlighted the alcohol consumption patterns among men who have sex with men. It was accurately worked by current literature to fulfill the study objectives. Suitable statistical methods are applied and interpretations are appropriate.

 For more clarification, reviewer’s comments are provided as follows:

**Abstract**

1. Regarding beer consumption, type of grouping should be mentioned precisely instead of “both groups”.
2. Clear description of the variables: “Together with their friends” and “at gathering of friends” is required at the start of article because repeated use of same word may cause a bit confusing.
3. In this study, only amount of alcohol consumption was found out by different time periods according to Table 4. However, it was mentioned that type and frequency were also identified by different time periods. Therefore, conclusion is necessary to be coincided with the finding.
4. There was no comparison of MSM with other specific groups in this article. Therefore, the proportion of current drinkers as well as binge drinking should not be concluded by the term, “higher”.

**Introduction**

1. Description of RDS in the second last paragraph is more appropriate.

**Methods**

1. As the pretesting was done in non-selected township, number of townships for purposive sampling from Yangon and Mandalay Cities should be mentioned.
2. Are there any other places of recruiting participants? Because “etc.” was added after drop-in-centers of International Non-governmental Organizations and beauty parlors in data collection.
3. Regarding three groups of MSM, similar word formatting should be used according to the literature because there is no consistency between abstract and operational definitions.
4. Types of alcoholic beverages mentioned in operational definitions section included “RUM” but following tables in results section were shown by “Whisky”. Therefore, it is better to explained about the discrepancy of usage if possible.
5. References should be cited for calculation of the number of standard drinks. Is it similar to amount of alcohol use?
6. In data management and analysis, statistical test for association and level of statistical significance are needed to be mentioned.

**Results**

1. In second paragraph, there was mentioning about the types of person that MSM drink with, the reasons and timings of consumption although Table 2 was shown amount and frequency of alcohol use.
2. The calculation of ethanol concentration by mean of grams in relation to mean amount of standard drinks should be explained.

Is the work clearly and accurately presented and does it cite the current literature?

Yes

If applicable, is the statistical analysis and its interpretation appropriate?

Yes

Are all the source data underlying the results available to ensure full reproducibility?

Yes

Is the study design appropriate and is the work technically sound?

Yes

Are the conclusions drawn adequately supported by the results?

Yes

Are sufficient details of methods and analysis provided to allow replication by others?

Yes

Reviewer Expertise:

Public Health, Epidemiology, Maternal and Reproductive Health, Infection Control, NCDs, Biostatistics, Environmental Health.

I confirm that I have read this submission and believe that I have an appropriate level of expertise to confirm that it is of an acceptable scientific standard.

# Response

**Published:** 2020-09-25

**Authors:** Kyaw-Min Htut

**Affiliations:** Department of Medical Research, Myanmar

**Thank you for your expert opinions and approval on our manuscript.**

****

**Comments from reviewer**

**Abstract**

 1. Regarding beer consumption, type of grouping should be mentioned precisely instead of “both groups”.

**Response:** Thanks for your comments and “both groups” means young age group (15-24 yrs) and adult age group (>24 yrs)

 2. Clear description of the variables: “Together with their friends” and “at gathering of friends” is required at the start of article because repeated use of same word may cause a bit confusing.

**Response: **Sorry for making you confusing. “Together with their friends” is one of the responses for the question "With whom they consumed alcohol". Similarly, “at gathering of friends” is the response for the reason of alcohol consumption.

 3. There was no comparison of MSM with other specific groups in this article. Therefore, the proportion of current drinkers as well as binge drinking should not be concluded by the term, “higher”.

**Response: **Proportion of current drinkers among MSM from current study was higher than the previous studies conducted among general population in Myanmar

**Methods**

 1. As the pretesting was done in non-selected township, number of townships for purposive sampling from Yangon and Mandalay Cities should be mentioned.

**Response: **The participants were recruited through Myanmar MSM network and it is not possible to mention the number of townships.

 2. Are there any other places of recruiting participants? Because “etc.” was added after drop-in-centers of International Non-governmental Organizations and beauty parlors in data collection.

**Response: **Others included the hotspot places of MSM were they usually gather such as home of famous beautician, moat and office of community based organization.

 3. Regarding three groups of MSM, similar word formatting should be used according to the literature because there is no consistency between abstract and operational definitions.

**Response: **I think three groups of MSM (Apwint, Apone and Thange) were used. 

 4. Types of alcoholic beverages mentioned in operational definitions section included “RUM” but following tables in results section were shown by “Whisky”. Therefore, it is better to explained about the discrepancy of usage if possible.

**Response:** Thank you for your comment. Whisky was missed to mention in the operational definition.
